# Associations of miR-499 and miR-34b/c Polymorphisms with Susceptibility to Hepatocellular Carcinoma: An Evidence-Based Evaluation

**DOI:** 10.1155/2013/719202

**Published:** 2013-09-26

**Authors:** Zhongxia Wang, Junhua Wu, Guang Zhang, Yin Cao, Chunping Jiang, Yitao Ding

**Affiliations:** ^1^Department of Hepatobiliary Surgery, Affiliated Drum Tower Hospital of Nanjing University Medical School, Nanjing, Jiangsu 210008, China; ^2^Jiangsu Province's Key Medical Center for Liver Surgery, Nanjing, Jiangsu 210008, China; ^3^School of Medicine, Nanjing University, Nanjing, Jiangsu 210093, China; ^4^Department of Hepatobiliary Surgery, Drum Tower Clinical Medical College of Nanjing Medical University, Nanjing, Jiangsu 210008, China

## Abstract

*Background*. Hepatocellular carcinoma (HCC) represents the sixth common cancer in the world. Single nucleotide polymorphisms (SNPs) in microRNA genes may be associated with susceptibility to HCC. Recently, several studies have reported possible associations of SNPs miR-499 T>C rs3746444 and miR-34b/c T>C rs4938723 with the risk of HCC. However the results are inconsistent and inconclusive. In this present study, we conducted a meta-analysis to comprehensively evaluate potential associations between the two SNPs and HCC susceptibility. *Methods*. Through a systematic literature search, 8-case-control studies involving 5464 subjects were identified and included in this meta-analysis. The association between the two common SNPs and HCC risk was estimated by pooled odds ratios (ORs) and 95% confidence intervals (95% CIs). Our results showed no significant association between rs3746444 and susceptibility to HCC, whereas variant genotypes of rs4938723 were associated with increased HCC risk in allele frequency model and heterozygous model (C versus T, OR = 1.11, 95% CI: 1.01–1.23, *P* = 0.04; TC versus TT, OR = 1.19, 95% CI: 1.03–1.37, *P* = 0.02). *Conclusions*. The current evidence did not support association between rs3746444 and HCC risk. SNP rs4938723 may be associated with susceptibility to HCC. Further well-designed studies are required to clarify the relationships between the two SNPs and HCC risk.

## 1. Introduction

Hepatocellular carcinoma (HCC) represents the most common primary malignancy of the liver. According to epidemiological survey, the prevalence of HCC ranks the sixth among all cancers. Although the diagnosis and treatment of HCC have significantly been improved in recent years, the prognosis remains poor. HCC accounts for approximately 700,000 cancer-related deaths per year, which ranks the third in global cancer statistics [1, 2]. The mechanism of hepatic carcinogenesis remains elusive. Chronic infection of hepatitis B and hepatitis C viruses (HBV and HCV) and subsequent liver injury-regeneration cycle are considered a major etiology of HCC [3]. However, only a small fraction of chronic viral hepatitis patients finally develop HCC while a considerable portion of HCC cases arise from livers without chronic hepatitis. This fact indicates that the carcinogenesis of HCC is a complex process with multiple factors involved [2, 4]. 

Recent studies indicate that genetic factors may play important roles in the development of HCC [4]. MicroRNAs (miRNAs) are a group of endogenous small noncoding RNA molecules with length of around 22 nucleotides. It is now clear that miRNAs function as important epigenetic regulators by negatively regulating the stability and transcriptional efficiency of messenger RNAs (mRNAs) [5]. miRNAs are involved in multiple pivotal processes of cancer development and progression including cell differentiation, proliferation, apoptosis, migration, and invasion [6, 7]. In recent years, the aberrance of miRNAs in cancers, including HCC, has been profiled and many oncogenic or tumor-suppressive miRNAs have been identified [8–10]. Among them, alterations of miRNA-coding genes may be of particular importance. Since one single miRNA regulates a wide spectrum of target genes, even small changes in the amount and function of a miRNA may result in extensive aberrance of tumor-promoter genes and tumor-suppressor genes and finally contribut to carcinogenesis [11].

 Single nucleotide polymorphisms (SNPs) are the most common genetic variations in population. Numerous SNPs in or around miRNA-coding genes have been identified, and their roles in human cancer have been implicated [12, 13]. Regulated by p53, both miR-499 and miR-34b/c are recognized as important factors in the process of carcinogenesis, apoptosis, and metastasis [14–16]. Recently, several molecular epidemiological studies have investigated potential associations of common SNPs rs3746444 in miR-499 [17–21] and rs4938723 in the promoter region of pri-miR-34b/c gene [22–24] with risk of HCC. Unfortunately, the results in these studies are controversial and inconclusive. Since individual study may be underpowered and biased for effective evaluation of potentially slight effects of the two functional polymorphisms due to relatively limited study population, a meta-analysis was conducted to achieve more comprehensive estimation of associations between the two common SNPs and susceptibility to HCC with up-to-date evidence.

## 2. Methods

### 2.1. Literature Search Strategy

A comprehensive computerized literature search was conducted in multiple online databases including PubMed, EMBASE, ISI Web of Science, the Cochrane Library, China National Knowledge Infrastructure (CNKI), Wanfang Database, and VIP Info database with the combination of the following search terms: “miR-499,” “miR-34,” “rs3746444,” “rs4938723,” “hepatocellular carcinoma,” “liver cancer,” “hepatoma,” and “HCC” to identify potentially relevant studies. The literature search was performed by two independent investigators (Zhongxia Wang and Junhua Wu, last update: July 15, 2013). Publication date, language, full article, or abstract was not restricted in our search. The reference lists of eligible studies were manually searched as a complement to electronic search. The results of search were crosschecked, and final consensus was reached between the two investigators.

### 2.2. Study Selection

**Figure 1:**
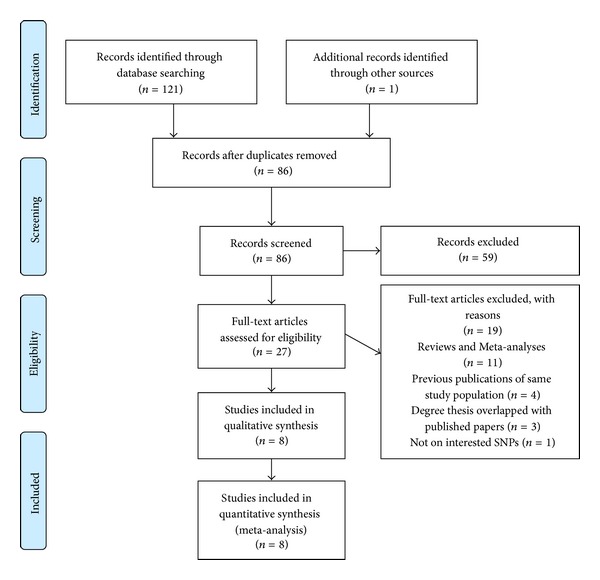
Flow diagram of literature search and selection.

Studies in our meta-analysis had to meet all of the following criteria: (1) case-control design; (2) evaluating the association between SNPs miR-499 T>C (rs3746444) and/or miR-34b/c T>C (rs4938723) and susceptibility to HCC; (3) being studied on human beings; (4) sufficient data of allele and genotype frequencies of SNPs being provided for the estimation of odds ratios (ORs) and 95% confidence intervals (95% CIs); (5) only the most recent study being included if serial studies of the same population were reported; and (6) methodological design being proper. Methodology of potential relevant case-control studies was evaluated using the following criteria: (1) the demographic data were comparable between case and control groups; (2) the diagnosis of HCC was determined with proper diagnostic methods; (3) appropriate methods were employed to determine genotypes; and (4) proper statistical methods were used. The details of study search and selection were provided in Figure 1 by flow diagram.

### 2.3. Data Abstraction

 Data were extracted from eligible studies by two independent investigators (Zhongxia Wang and Junhua Wu). The extracted data included the authors' name, publication date, origin of the study, ethnicity of studied population, methods of genotyping, allele, and genotype frequencies in both case and control groups. The two investigators crosschecked the results of data abstraction. When different results were obtained, they repeated data extraction of the specific study and discussed them to reach mutual agreement. If disagreement still existed, two senior investigators (Yitao Ding and Chunping Jiang) were invited to discussion until consensus was reached.

### 2.4. Data Synthesis and Statistical Analysis

Chi-square test was used to test whether the distribution of genotypes in control group deviated from HWE. ORs and corresponding 95% CIs were calculated to evaluate the strength of associations between SNPs and the risk of HCC under the following genetic models: (1) allele frequency (C versus T); (2) heterozygous model (TC versus TT); (3) homozygous model (CC versus TT); (4) dominant model (TC + CC versus TT); and (5) recessive model (CC versus TT + TC). Heterogeneity among included studies was evaluated by Cochrane *Q*-test and *P* > 0.10 indicated that no significant statistical heterogeneity existed [25]. Pooled ORs and 95% CIs were generated to estimate the effect of rs3746444 and rs4938723 on susceptibility to HCC. If no significant heterogeneity was detected, the pooled ORs were generated by fixed-effects model (Mantel-Haenszel method) [26]. Otherwise, the random-effects model (DerSimonian-Laird method) was used to estimate pooled statistics [27]. The significance of pooled ORs was determined by *Z*-test. A *P* value less than 0.05 was considered as statistically significant. Corresponding forest plots were generated to show the results of meta-analyses. 

Sensitivity analysis was conducted by excluding individual study in turn to observe the change in heterogeneity test and the significance of pooled ORs. For miR-499 T>C (rs3746444), subgroup analysis was carried out using data from Asian ethnicity, HBV-infected cases and studies within HWE. All of the data synthesis and statistical analysis were performed using Review Manager 5.2.5 software (Copenhagen: The Nordic Cochrane Centre, The Cochrane Collaboration, 2012).

## 3. Results

### 3.1. Study Characteristics

As shown in Figure 1, 121 potentially relevant articles were retrieved by computerized database search. Reference list examination further identified another relevant study. After the removal of duplicates, 86 records were screened by reviewing titles and abstracts. According to inclusion criteria, 59 records were excluded. The remaining 27 full-text articles were retrieved and assessed. Eight eligible studies were finally included in this meta-analysis [17–24].

Characteristics of included studies are shown in Table 1. Five studies on miR-499 T>C rs3746444 involving 852 cases and 1191 controls were included in this meta-analysis [17–21]. For miR-34b/c T>C rs4938723, a total of three studies reported potential association between this SNP and the risk of HCC with evidence from 1672 cases and 1749 controls [22–24]. All of the studies except the report from Akkiz et al. [17] were carried out in Asian population. The method of genotyping included polymerase chain reaction-restriction fragment length polymorphism (PCR-RFLP) and real-time polymerase chain reaction. For the studies from Akkiz et al. [17] and Zou and Zhao [21], genotype distribution in control group deviated from HWE. Genetic distributions in control groups of the rest studies conformed to HWE.

### 3.2. Lack of Association between miR-499 T>C rs3746444 and Susceptibility to HCC

 The association between rs3746444 and the risk of HCC was analyzed using data from five independent studies [17–21]. The results of this meta-analysis were summarized in Table 2. Significant statistical heterogeneity was detected in all of the genetic models except heterozygous model. Therefore the random-effect model was employed to calculate the pooled ORs. To our surprise, the results demonstrated no significant association between rs3746444 and the risk of HCC in any genetic model tested. Sensitivity analysis revealed that the study from Xiang et al. [19] was the main source of statistical heterogeneity since the significance of *Q*-test became negative after the exclusion of this study. However, there was still no obvious association between rs3746444 and susceptibility to HCC in the remaining studies with less heterogeneity.

 Subgroup analyses were conducted in Asians, HBV infected HCC patients and in studies that conformed to HWE. Similarly, miR-499 T>C rs3746444 was not associated with HCC susceptibility in all genetic model analyzed in Asians. Four studies provided genotypes in HBV-associated HCC patients [17–19, 21]. Subgroup analysis showed that SNP rs3746444 did not modify the risk of HCC in the patients with chronic HBV infection. Likewise, for studies within HWE, which were of less selection bias, consistent negative results were confirmed. 

### 3.3. Association between miR-34b/c T>C rs4938723 and Susceptibility to HCC

**Figure 2:**
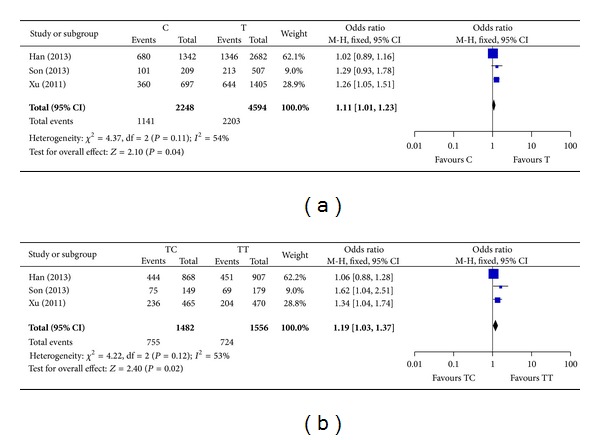
Forest plots of meta-analysis of association between rs4938723 and the risk of HCC. (a) Meta-analysis under allele frequency model. (b) Meta-analysis under heterozygous model. The blue squares and corresponding horizontal lines indicate odds ratio of individual study. The area of the squares reflects weight of indicated study. The black filled diamond represents pooled odds ratio and 95% confidence interval.

Three studies reported potential association between rs4938723 and HCC risk with evidence from 1672 cases and 1749 controls [22–24]. All of these studies were carried out in Asian population. Results of this meta-analysis were shown in Table 3. Fixed-effect model was used in most genetic models to estimate pooled ORs and 95% CIs since no significant heterogeneity was detected except for dominant model. Significant associations of rs4938723 with the risk of HCC were observed in allele frequency model and heterozygous model (Figure 2). Compared with T allele, C variant of rs4938723 was associated with a higher risk of HCC (OR = 1.11, 95% CI: 1.01–1.23, *P* = 0.04). In heterozygous model, carriers of TC genotype were more susceptible to HCC compared with TT carriers (OR = 1.19, 95% CI: 1.03–1.37, *P* = 0.02). A trend of association was also observed in dominant model although it did not reach statistical significance with a marginal *P* value of 0.06 (OR = 1.25, 95% CI: 0.99–1.58). No significant association was demonstrated in homozygous model and recessive model. 

## 4. Discussion

 Despite significant advancements in the research of HCC, the detailed etiology of this fatal disease remains elusive. Besides well-known risk factors, such as viral hepatitis, alcohol abuse, and nonalcoholic fatty liver disease (NAFLD), genetic factors may also contribute to the development of HCC [4, 28]. Identification of genetic biomarkers of HCC susceptibility may be extremely valuable in facilitating early diagnosis and discovering molecular targets for personalized treatment. As important epigenetic regulators, miRNAs are crucial in the process of liver carcinogenesis by acting as either oncogenes or tumor-suppressor genes [29]. SNPs represent the most common genetic polymorphisms in human genome. Through altering the expression, stability, and function of miRNAs, SNPs may indirectly affect a wide range of cancer-related genes and thus play important role in individual's susceptibility to HCC [30, 31]. Therefore, SNPs of miRNA-coding genes may serve as genetic biomarkers of HCC risk. miR-499 and miR-34b/c are regulated by tumor-suppressor gene p53 and may participate in hepatic carcinogenesis [15, 16]. Recently, two functional SNPs miR-499 T>C rs3746444 and miR-34b/c T>C rs4938723 have been reported to associate with HCC susceptibility [17–24], indicating potential value of these SNPs in risk screening of HCC. However results from these studies are controversial and inconclusive, likely because of the limited sample size of individual study and potential selection bias. In this present meta-analysis, we systematically analyzed eight independent case-control studies and reevaluated the potential association of the two common SNPs with susceptibility to HCC.

 miR-499 participates in several crucial cancer-related biology processes such as apoptosis, cell migration, cell senescence, and inflammation [32–34]. Regulated by p53, miR-499 inhibits apoptosis by targeting calcineurin and dynamin-related protein-1 (Drp1). More importantly, Lafferty-Whyte et al. revealed that miR-499 functions as a prometastatic miRNA. It promotes the migration and invasion of colorectal cancer cells via regulating the expression of forkhead box O4 (FOXO4) and programmed cell death 4 (PDCD4) [34]. Together with miR-34c, this miRNA also regulates multiple types of cellular senescence induction [32]. miR-499 T>C polymorphism (rs3746444) results in an alteration from A:U pair to G:U mismatch in the stem region of miR-499, which reduces secondary structure stability and affects the process of miRNA maturation as well as binding to target mRNA [35]. Therefore, rs3746444 may contribute to susceptibility to cancer by regulating downstream genes. Positive associations between rs3746444 and the risks of various cancers have been reported [35–37]. Five studies demonstrated inconsistent results of association between this SNP and HCC risk. Three of them suggested significant association [18, 19, 21] while the rest studies reported no obvious association observed [17, 20]. 

 In this meta-analysis, we assessed possible association between SNP rs3746444 and susceptibility to HCC with evidence from 852 cases and 1191 controls. Our results elucidated that there lacks any association between rs3746444 and the risk of HCC in all of genetic models tested. Sensitivity analysis revealed that the study from Xiang et al. [19] was the main source of heterogeneity. However, the association remained negative even this study was excluded. To address effects of potential confounding factors on the results of this meta-analysis, we performed several subgroup analyses. We firstly conducted subgroup analysis in Asian population since the incidence of SNP may vary between different ethnicity. After that, studies conformed to HWE were synthesized to rule out selection bias. Stratified analysis by HBV infection status was also carried out to identify potential interaction between SNP and HBV infection. Consistently, no significant association was observed in all of the subgroup analyses. The results of this meta-analysis do not support a genetic association of rs3746444 with susceptibility to HCC. Sensitivity analysis and subgroup analyses confirmed the stability of the results.

 As members of the miR-34 family, miR-34b and miR-34c share a common primary transcript (pri-miR-34b/c). Induced by p53 in response to genotoxic stress, miR-34b/c are considered as tumor-suppressor miRNAs [15, 38]. Previous studies revealed that the miR-34 family acts as negative regulators of hundreds of oncogenes and subsequently induce cell cycle arrest or cell death [15, 39]. Gene therapy using miR-34 as target molecule was proven effective for HCC in animal models [38]. In patients, downregulation of miR-34b/c by CpG islands methylation in the promoter region was frequently observed in cancers from various histological sources [40–45]. SNP rs4938723 (T>C) is located within CpG islands in the promoter of pri-miR-34b/c and may participate in epigenetic silence of miR-34b/c. Furthermore, this T>C variant is also predicted to affect the binding of GATA-X transcription factors to the promoter region of pri-miR-34b/c gene [22]. Hence rs4938723 may alter the promoter transcription activity, affect the expression level of miR-34b/c, and subsequently cause change in susceptibility to cancer [18]. Indeed, this SNP is reported to be associated with susceptibility to colorectal cancer [46], nasopharyngeal carcinoma [47], breast cancer [48], and HCC [22–24]. Although all studies on HCC demonstrated association between rs4938723 and HCC risk, the genetic models with statistical significance were inconsistent.

 In the present study, we systematically evaluated the potential association of rs4938723 with susceptibility to HCC with data from 1672 cases and 1749 controls. Statistically significant associations were demonstrated in analyses under allele frequency (C versus T) and heterozygous model (TC versus TT). Genetic variant T>C increases the risk of HCC in these two genetic models. A trend of elevated risk of HCC was observed in dominant model (TC + CC versus TT) although this trend did not reach statistical significance with a marginal *P* value of 0.06. Importantly, obvious heterogeneity existed in the estimation of dominant model. However, limited number of eligible studies and lack of detailed information prevented us from exploring the source heterogeneity by subgroup analysis. Possible association between rs4938723 and the risk of HCC under dominant model should not be rule out.

 To the best of our knowledge, this is the first meta-analysis evaluating the association between miR-34b/c T>C rs4938723 and susceptibility to HCC. This is also the latest study assessing potential association between miR-499 T>C rs3746444 and HCC risk with the most evidence, though the conclusion was in agreement with previous meta-analyses [49–51]. However, the results of this study should be interpreted cautiously due to several limitations. Firstly, even after synthesizing all available data, the sample size remains relatively small. This may limit the power of this study to detect potential slight effects of these two SNPs on HCC susceptibility. The limited number of included studies also prevented us from evaluating publication bias by funnel plot. It should be noted that potential publication bias may introduce confounders and the conclusion might deviate from true effect. Moreover, heterogeneity was found significant in most analyses of rs3746444. Although sensitivity analysis and subgroup analyses confirmed the stability of our results, it should be acknowledged that inherent heterogeneity may distort the true effect and may reduce the reliability of the results. Finally, there was no sufficient data regarding age, gender, smoking, alcohol consumption, the status of fatty liver diseases, and so forth, for clarifying their effects on HCC susceptibility and interactions with SNPs. These factors may confound with true effects of SNPs and conceal or exaggerate the possible associations.

 In conclusion, the SNP rs4938723 in the promoter region of pri-miR-34b/c gene is associated with elevated susceptibility to HCC. However, our meta-analysis does not support association between rs3746444 and the risk of HCC. Further well-designed studies with larger sample size are required to verify our findings. Functional studies and interactions of these two SNPs with other predisposing or protective factors of HCC will be of great interest. The value of SNP rs4938723 as a genetic biomarker for early diagnosis or a molecular target for novel therapy of HCC is also worthy of investigating.

## Figures and Tables

**Table 1:** Characteristics of included studies.

Author	Year	Country	Ethnicity	SNP	Genotyping methods	*P* for HWE	Case genotype			Control genotype		
							TT	TC	CC	TT	TC	CC
Akkiz	2011	Turkey	Caucasian	rs3746444	PCR-RFLP	0.036	45	87	90	47	93	82
Kim	2012	Korea	Asian	rs3746444	PCR-RFLP	0.278	109	47	3	120	74	7
Xiang	2012	China	Asian	rs3746444	PCR-RFLP	0.284	36	40	24	54	36	10
Zhou	2012	China	Asian	rs3746444	PCR-RFLP	0.620	141	41	4	371	100	12
Zou	2013	China	Asian	rs3746444	PCR-RFLP	0.005	136	44	5	123	48	14
Xu	2011	China	Asian	rs4938723	Real-time PCR	0.647	204	236	62	266	229	54
Son	2013	Korea	Asian	rs4938723	PCR-RFLP	0.371	69	75	13	110	74	17
Han	2013	China	Asian	rs4938723	PCR-RFLP	0.183	451	444	118	456	424	119

SNP: single nucleotide polymorphism; *P* for HWE: *P* value for Hardy-Weinberg equilibrium (HWE) calculated by Chi-square test. *P* < 0.05 indicates deviation of genotype distribution from HWE.

**Table 2:** Meta-analysis of the association between SNP miR-499 (T>C) rs3746444 and susceptibility to HCC.

Genetic model	Population	OR	95% CI	*Z*	*P*	*P*-h
Allele frequency C versus T	Overall	1.01	0.72–1.43	0.06	0.95	0.0008
	Asian	0.99	0.62–1.59	0.04	0.97	0.0004
	HBV infected	0.96	0.59–1.56	0.16	0.87	0.001
	HWE	1.13	0.64–2.01	0.43	0.67	0.001

Heterozygous model TC versus TT	Overall	0.96	0.77–1.19	0.39	0.70	0.23
	Asian	0.95	0.75–1.21	0.38	0.70	0.13
	HBV infected	0.83	0.54–1.28	0.83	0.40	0.09
	HWE	1.04	0.66–1.63	0.17	0.86	0.07

Homozygous model CC versus TT	Overall	0.96	0.44–2.09	0.10	0.92	0.006
	Asian	0.87	0.27–2.85	0.23	0.82	0.003
	HBV infected	1.04	0.45–2.43	0.10	0.92	0.04
	HWE	1.24	0.36–4.34	0.34	0.73	0.02

Dominant model TC + CC versus TT	Overall	1.00	0.71–1.42	0.00	1.00	0.02
	Asian	0.99	0.63–1.55	0.03	0.97	0.009
	HBV infected	0.90	0.53–1.53	0.40	0.69	0.01
	HWE	1.12	0.63–1.99	0.37	0.71	0.009

Recessive model CC versus TT + TC	Overall	0.97	0.50–1.88	0.08	0.93	0.02
	Asian	0.86	0.30–2.45	0.28	0.78	0.009
	HBV infected	1.23	0.86–1.75	1.15	0.25	0.12
	HWE	1.22	0.43–3.47	0.37	0.71	0.06

OR: odds ratio; 95% CI: 95% confidence interval; *Z*: *Z* value for *Z*-test; *P*: *P* value for *Z*-test; *P*-h: *P* value for *Q*-test; HBV infected: subgroup analysis in hepatitis B virus (HBV) infected cases. HWE: only studies that conform to Hardy-Weinberg equilibrium were included in this subgroup analysis.

**Table 3:** Meta-analysis of the association between SNP miR-34b/c (T>C) rs4938723 and susceptibility to HCC.

Genetic model	OR	95% CI	*Z*	*P*	*P*-h
Allele frequency C versus T	1.11	1.01–1.23	2.10	0.04	0.11
Heterozygous model TC versus TT	1.19	1.03–1.37	2.40	0.02	0.12
Homozygous model CC versus TT	1.15	0.92–1.44	1.22	0.22	0.28
Dominant model TC + CC versus TT	1.25	0.99–1.58	1.85	0.06	0.09
Recessive model CC versus TT + TC	1.06	0.86–1.31	0.55	0.58	0.49

OR: odds ratio; 95% CI: 95% confidence interval; *Z*: *Z* value for *Z*-test; *P*: *P* value for *Z*-test; *P*-h: *P* value for *Q*-test.
